# Prospective analysis of clinical, cognitive and functional impairments among older adults following a diagnosis of covid

**DOI:** 10.1002/alz.089914

**Published:** 2025-01-03

**Authors:** Fabiana Carla Matos da Cunha Cintra, Marco Túlio Cintra, Estevão Alves Vallle, Henrique Dias Furtado De Souza, Daniella Cristina Brites Almeida, Iza de Faria Fortini, Bernardo de Mattos Viana, Maria Aparecida Camargos Bicalho

**Affiliations:** ^1^ Federal University of Minas Gerais, Belo Horizonte, Minas Gerais Brazil; ^2^ Clínica Mais 60, Belo Horizonte, Minas Gerais Brazil; ^3^ UFMG, Belo Horizonte Brazil; ^4^ Elderly Psychiatry and Psychology Extension Program (PROEPSI), Faculdade de Medicina, Universidade Federal de Minas Gerais, Belo Horizonte Brazil; ^5^ National Institute of Science and Technology Neurotec R (INCT‐MM), Faculdade de Medicina, Universidade Federal de Minas Gerais, Belo Horizonte Brazil; ^6^ Universidade Federal de Minas Gerais, Belo Horizonte Brazil; ^7^ INCT – NeuroTecR and CTMM, Belo Horizonte, Minas Gerais Brazil

## Abstract

**Background:**

Post‐Covid syndrome has been associated to enduring impairments in functionality, cognition, mood and physical capabilities among older adults.

**Methods:**

The objective was to prospectively evaluate clinical, cognitive and functional impairments in elderly people at 3 and 12 months after the diagnosis of Covid‐19. Prospective cohort study of participants aged 60 years and over after a Covid‐19 diagnosis. The project was approved by the UFMG’s ethics committee. The assessment protocol included the scales Alzheimer’s Disease Cooperative Study ‐ Activities of Daily Living (ADCS‐ADL), Canadian Occupational Performance Measure (COPM), Clinical Frailty Scale (CFS), Mattis Dementia Rating Scale (DRS), Post‐Traumatic Stress Disorder Checklist – Civilian Version, Geriatric Depression Scale ‐ 15 items, Charlson Comorbidity Index, modified Medical Research Council for dyspnea and Fatigue Severity Scale. The statistical analysis included Paired Samples T Test for continuous variables, the Mcnemar Test for categorical variables and the Sign Test for ordinal variables.

**Results:**

Twenty‐eight participants with a mean age of 69.46 ± 7.09 years and 8.04 ± 4.76 years of education were evaluated after 3 months (T1) and 12 months (T2). Women represented 56.6% and 82.1% of participants were hospitalized in the acute phase. There was an increase in frailty from 7.2% of frail people prior to covid diagnosis, to 39.3% at T1 (P = 0.004) and 32.2%, at T2 (p = 0.016) assessed by CFS. Functional recovery was not statistically significant (ADCS‐ADL ‐ P = 0.083), however, the subitem satisfaction and concerning functional performance assessed by COPM improved (P = 0.012). At (T1) 57.2% of the sample presented a diagnosis of MCI and 17.9% of dementia, while at (T2) 60.7% and 17.9%, respectively (P = 1.00). However, the DRS’s conceptualization domain showed statistical significance improvement [P = 0.011]. Dyspnea symptoms had a significant improvement: from 82.2% with grade II to IV dyspnea at (T1) to 50% at T2 (P = 0.031). No changes were observed in fatigue, depressive symptoms and post‐traumatic stress disorder.

**Conclusion:**

This study showed that symptoms such as fatigue, cognitive and functional changes may persist in older adults with following the diagnosis of covid. However, there was a significant improvement in dyspnea, in the DRS’s conceptualization domain and satisfaction in relation to functional performance after 12 months of follow‐up.

